# Ultrasonic-assisted top-down preparation of NbSe_2_ micro/nanoparticles and hybrid material as solid lubricant for sliding electrical contact

**DOI:** 10.1016/j.ultsonch.2021.105491

**Published:** 2021-02-10

**Authors:** Rong Qu, Xiaoqin Wen, Yamei Zhao, Tingmei Wang, Ruiqing Yao, Jinjun Lu

**Affiliations:** a Key Laboratory of Synthetic and Natural Functional Molecule of Ministry of Education, College of Chemistry and Materials Science, Northwest University, Xi’an 710127, China; b State Key Laboratory of Solid Lubrication, Lanzhou Institute of Chemical Physics, Chinese Academy of Sciences, Lanzhou 730000, China

**Keywords:** NbSe_2_, Exfoliation, Ultrasonic-assisted, Ageing, Electrical contact

## Abstract

2H-NbSe_2_ single crystal flake (ca. 2 × 2 × 0.5 mm in size) by chemical vapor transport is employed as the precursor for a top-down preparation of NbSe_2_ micro/nanoparticles by two kinds of processes, i.e. (1) mechanical exfoliation; (2) ultrasonic-assisted exfoliation in ethanol without ageing and with ageing for 210 days. NbSe_2_ micro/nanoparticles are applied on top of a Cu disk by a drop-casting process and the tribological property in sliding against a Cu pin under sliding electrical contact is investigated at room temperature. Mechanical exfoliation produces NbSe_2_ microplatets with typical sizes of 1 μm to 30 μ m with a thickness less than 2 μm. Ultrasonic-assisted exfoliation without aging facilitates the formation of NbSe_2_ micro/nanoplatets with sizes of 0.1 μm to 25 μm and nano-whiskers with 100 nm in diameter and 1 ~ 3 μm in length, but Nb_2_O_5_ and Se are also found on the basis of XPS results. Prolonged aging of the suspensions modifies the morphology by converting platets and whiskers into corrugated floccules (hybrid material), which are composed of Nb_2_O_5_, Se, NbSe_2_, and graphene. Notably, NbSe_2_ micro/nanoparticles by ultrasonic-assisted exfoliation without ageing exhibit an excellent lubricating property with low friction coefficient (0.3), mild wear, and longer wear lifetime (120 min) than that of mechanical exfoliated NbSe_2_ microplatets (10 min). The wear lifetime for the aged NbSe_2_ micro/nanoparticles can be as long as 504 min and are 4.2 times of the sample without aging, which can be a good solid lubricant for sliding electrical contact

## Highlights

• NbSe_2_ micro/nanoparticles were achieved by ultrasonic-assisted exfoliation.
• Prolonged ageing process modifies the morphology and chemical composition.
• The excellent lubricating property with low friction coefficient and longer lifetime is obtained.

## 1 Introduction

Metal-matrix self-lubricating composites (MMSC) containing metal chalcogenides (e.g. MoS_2_, NbSe_2_) as solid lubricant fabricated by powder metallurgy have been widely applied in sliding electrical contact (e.g. brush-slip) for many years [1], [2]. In most cases, MoS_2_ rather than NbSe_2_ is the main constitution of the composite due to its good lubricity. In specific, the excellent lubricity of MoS_2_ in various environments (e.g. vacuum) can be attributed to its excellent ability of transfer film-forming. However, the application of MoS_2_ is greatly limited by its poor electrical conductivity (0.118 S). In contrast, NbSe_2_ exhibits an excellent electrical conductivity (1.87 × 10^5^ S) but a relatively poor lubricity due to its poor film-forming ability.

Recently, NbSe_2_ film (c.a. 1.5 μ m in thickness) by radio frequency magnetron sputtering shows good tribological property and good electrical conductivity [3]. This provides an alternative route for NbSe_2_ as solid lubricant coatings for electrical contact. Inspired by this result, NbSe_2_ coatings on electrically conducted metal (e.g. Cu and Cu alloys) either by mechanical exfoliation and transfer or by solution-based techniques (e.g. drop-casting, spraying) are also possible processes for protecting electrical contact from severe wear. There are some good examples for graphene as a protecting coating by mechanical exfoliation and transfer [4] and drop-casting [4], [5], [6] for electrical contact. This kind of coating has several advantages over MMSC containing graphene [7], [8], i.e. low consumption of solid lubricant, no risk of poor mechanical strength, and fast and efficient process; it also has much lower thickness than the sputtering film in reference [3] and therefore it produces much less wear debris. In principle, drop-casting is superior to mechanical exfoliation and transfer in considering the efficiency and reliability of the process. Until now, NbSe_2_ coating by solution-based techniques for electrical contact remains unstudied.

For solution-based process, NbSe_2_ as a solid loading in a volatile solvent should be prepared as precursor for drop-casting or spraying. NbSe_2_ nanoparticles, i.e. 2D nanoplates and 1D nanowires by a solution-based synthesis [9] and nanoplates and nanosheets by solid state synthesis [10], [11], [12] can be used for solution-based process. This is supported by the fact that ultrasonic-assisted preparation of nanoparticles of a variety of materials has come a long way [13], [14], [15], [16], [17], [18] in recent years and proves to be an efficient way for exfoliation of 2D materials [19] and layered materials [14], [16]. For ultrasonic-assisted preparation, its excellent ability to exfoliate 2D materials makes it possible for a top-down preparation of NbSe_2_ micro/nanoparticles or even few layers NbSe_2_ in organic solvent using single crystal NbSe_2_ flake as a precursor. As a matter of fact, NbSe_2_ nanotubes and nanofibers have been successfully prepared by chemical vapor transport (CVT) [20] and CVT large flake with a size of 10 mm is commercially available by HQ Graphene.

It is a good choice using a top-down preparation of NbSe_2_ micro/nanoparticles via. ultrasonic-assisted exfoliation of single crystal NbSe_2_ flake when considering the following three issues, i.e. (1) application of NbSe_2_ micro/nanoparticles to the working surface of electrical contact, (2) chemical modification of NbSe_2_ micro/nanoparticles, and (3) the surface topography effect on the tribological property. For issue 1, it should be noted that thin film approach with preapplied liquid lubricant on member of electrical contact has been widely used due to convenience and effectiveness. In specific, clean metal sample is coated with liquid lubricant by applying dilute lubricant solutions in a volatile carrier, e.g. 1,1,1-trichloroethane, ethanol [21]. Likewise, the as-received NbSe_2_ micro/nanoparticles in organic solvent (e.g. ethanol) can be feasibly applied to the member of electrical contact in similar processes (e.g. drop-casting). For issue 2, ultrasonic-assisted exfoliation of single crystal NbSe_2_ flake makes it possible for improved tribological property via. chemical modification of NbSe_2_ micro/nanoparticles in various ways (e.g. ageing, surfactant). For issue 3, from the viewpoint of tribology, both few layers NbSe_2_ and NbSe_2_ micro/nanoparticles are promising solid lubricant for electrical contact. The latter one (one-pot without centrifugation) is no doubt much more easily available than the former one and is more suitable for rough surfaces (e.g. abraded, surface textured). For example, few layer 2D materials are not engaged in sliding on a surface texture on a several tens of micrometer in size and this makes 2D material ineffective as a solid lubricant on such surface textures [22].

Herein, by using single crystal NbSe_2_ flake as precursor, NbSe_2_ micro/nanoparticles have been successfully synthesized by mechanical exfoliation and ultrasonic-assisted exfoliation in ethanol with or without ageing. The phase composition, surface chemistry, and morphology of NbSe_2_ micro/nanoparticles via. ultrasonic-assisted exfoliation without and with ageing of the suspensions are investigated in details. Furthermore, the lubricating property of NbSe_2_ micro/nanoparticles is performed and the wear mechanism is briefed.

## 2 Experimental

### 2.1 Materials

#### 2.1.1 Single crystal 2H-NbSe_2_

Single crystal 2H-NbSe_2_ wafer with a purity of >99.995% prepared by chemical vapor transport is commercially available from HQ graphene Holland. It is the “mother” for NbSe_2_ micro/nanoparticles through exfoliation.

#### 2.1.2 Mechanical exfoliation and ultrasonic-assisted exfoliation

A NbSe_2_ flake (ca. 2 × 2 × 0.5 mm in size) is carefully torn from the “mother” wafer for the following two kinds of exfoliation processes, i.e. (1) mechanical exfoliation by tape, and (2) ultrasonic-assisted exfoliation in organic solvent. Ethanol is selected in this study because it is also a good solvent commonly used in drop-casting process [4], [5], [6], [21].

Mechanical exfoliation of the NbSe_2_ flake is conducted using a specially designed tape in a standard way available from the supplier. No further treatment (e.g. sedimentation, centrifugation) is conducted for screening NbSe_2_ micro/nanoplatets of various sizes. Finally, NbSe_2_ micro/nanoplatets on tape are transferred onto Cu disk and ready for characterization and tribological property.

Ultrasonic-assisted exfoliation is conducted using neither a microtip nor a water–ice bath. As such, temperature rise of the suspension and thermal decomposition of NbSe_2_ are not intentionally avoided. A 0.57 mg/ml NbSe_2_ flake in ethanol (3 ml) is sealed in a centrifugal tube. Then the centrifugal tube is treated in a SYU4-180D ultrasonic bath using a power of 100 W successively for 10 h and is cooled down to room temperature. Finally, the suspensions are obtained without any further treatment (e.g. sedimentation, centrifugation). In addition, ageing of the suspensions up to 210 days is conducted at room temperature in lucifuge.

#### 2.1.3 NbSe_2_ micro/nanoparticles on Cu by drop-casting

NbSe_2_ micro/nanoparticles for the tribological tests are deposited on a Cu disk by drop-casting process. The suspensions in section 2.1.2 are the precursor for drop-casting. A 60 μl suspension is applied for one side of a ϕ25 mm Cu disk. The surface of Cu disk is abraded using abrasive paper and ultrasonically cleaned before use. After complete evaporation of ethanol, NbSe_2_ micro/nanoparticles on a ϕ25 mm Cu disk is ready for tribological tests.

### 2.2 Tribological tests

Sliding electrical tests are conducted on an MFC4000 tribo-meter with a pin-on-disk configuration. A copper pin (3 mm in diameter and 15 mm in length with a hemispherical tip of 3 mm) sliding on a copper disk (25 mm in diameter and 3 mm in thickness) are used. Experimental details can be found elsewhere [21]. Test conditions are: 0.76 mm/s for sliding speed, 3 N for normal load, 20–25 °C for room temperature. The selection of sliding speed and normal load is based on the condition of severe adhesive wear occurred for unlubricated Cu-on-Cu contact. Under the same condition, the presence of NbSe_2_ micro/nanoparticles can greatly reduce adhesive wear.

### 2.3 Characterization

The worn surfaces of pin and disk are carefully examined and characterized. The morphology is observed using a ZEISS SIGMA field emission scanning electron microscopy (FESEM) with energy dispersive spectroscopy, and GZF2.0 transmission electron microscopy (TEM) of FEI Electron Optics. Raman (HR Evolution) is used to characterize NbSe_2_ micro/nanoparticles. XPS is used for determination of chemical states of selected elements of NbSe_2_ micro/nanoparticles on PHI 5000VersaProbe III.

## 3 Results and discussion

### 3.1 Mechanically exfoliated NbSe_2_ microplatets

**Fig. 1:**
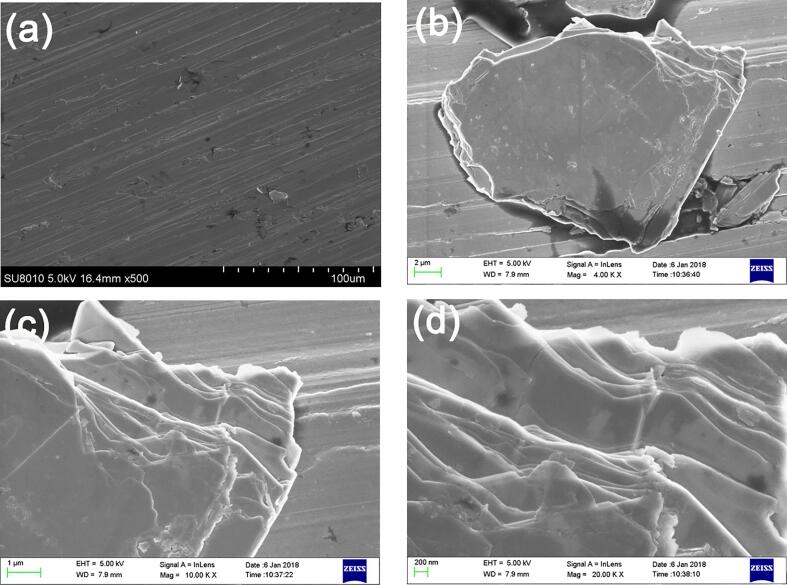
FESEM micrographs of (a) mechanically exfoliated NbSe_2_ microplatets transferred to Cu disk (one time transfer); (b) an individual microplatet, (c,d) the edge of an individual microplatet in (b).

Mechanical exfoliation of 2H-NbSe_2_ flake produces NbSe_2_ microplatets of various sizes (typical size of 1 μm to 30 μ m with a thickness less than 2 μ m) and it takes at least 10 times to transfer adequate amount of NbSe_2_ microplatets onto Cu disk for the tribological tests, see Fig. 1a. The individual microplatet is a layered crystal with irregular edge (Fig. 1b). Raman spectrum in Fig. S1 justify the microplatets as NbSe_2_ by 230 cm^−1^ for Se-Se (A^1^_g_) and 237 cm^−1^ for Nb-Se (E^1^_2g_). Unlike XRD pattern of 2H-NbSe_2_ flake (Fig. S2a), XRD pattern suggest the presence of only (0 0 2) plane for sample by mechanical exfoliation, which is similar to those of ultrasonic-assisted exfoliation without ageing and after ageing for 15 days (Fig. S2b). Evidences for delamination and cleavage are presented in Fig. 1c and Fig. 1d, respectively. No corrugation on the edge of microplatet is observed.

**Fig. 2:**
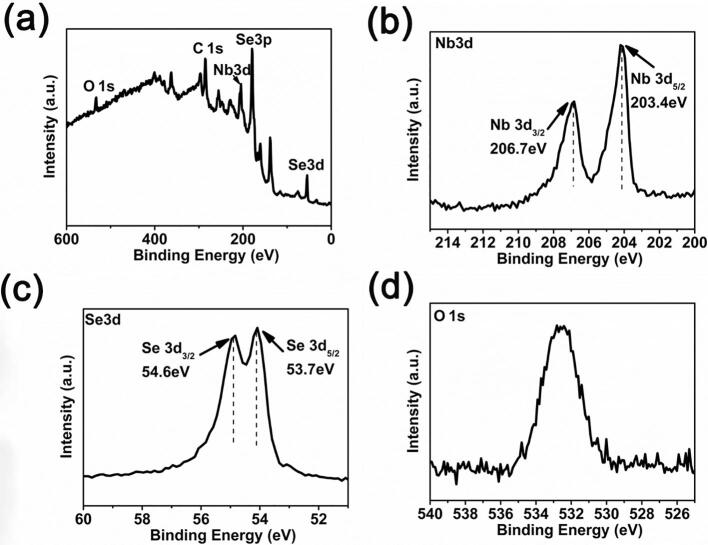
XPS spectra of (a) survey, (b) Nb3d, (c) Se3d, (d) O1s after being sputtered for 12 s using Ar^+^ ion.

XPS spectra in Fig. 2 show that very mild oxidation on the surface of mechanically exfoliated NbSe_2_ microplatets and the naturally occurred oxide can be readily removed by Ar^+^ ion sputtering for 12 s. The presence of N1s at 400 eV in Fig. 1a is attributed by the carrying gas used in chemical vapor transport process.

### 3.2 Ultrasonic-assisted exfoliation

#### 3.2.1 Without ageing

**Fig. 3:**
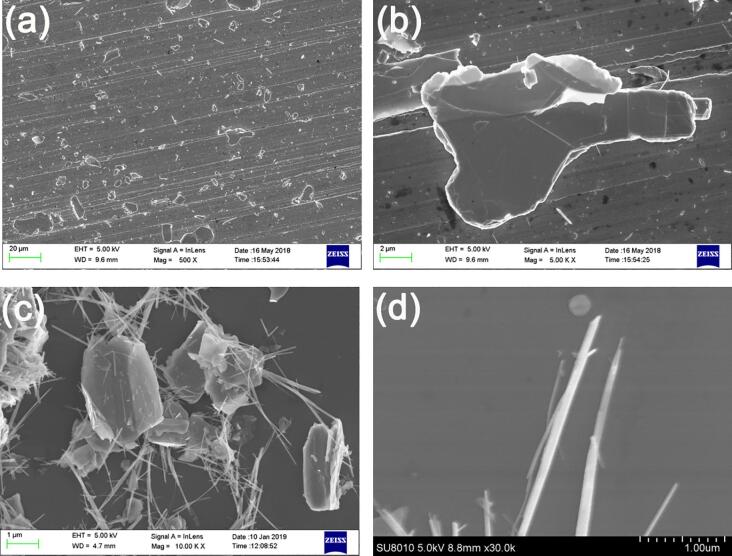
FESEM micrographs of (a) ultrasonic-assisted exfoliated NbSe_2_ microparticles transferred to Cu disk; (b) an individual microplatet with cracks on the surface, (c) agglomerated micro/nanoplatets and nanowhiskers, (d) nanowhiskers.

Ultrasonic-assisted exfoliation of 2H-NbSe_2_ flake produces micro/nanoparticles in two kinds of morphologies, i.e. (1) micro/nanoplatets and (2) nanowhiskers. By using as-received NbSe_2_-ethanol suspensions after ultrasonic treatment, the drop-casting process enables a homogeneous distribution of micro/nanoplatets and nanowhiskers on Cu disk, see Fig. 3a. The particle sizes of micro/nanoplatets range from 0.1 μm to 25 μm with a thickness less than 1 μm (Fig. 3b), which is smaller than that of mechanical exfoliated ones (Fig. 1b). No corrugation on the edge of micro/nanoplatets is observed. Agglomeration of micro/nanoplatets and nanowhiskers can be observed, see Fig. 3c. The average diameter of nanowhiskers is ca. 100 nm and the length is from 1 to 3 μm, see Fig. 3b and 3d.

**Fig. 4:**
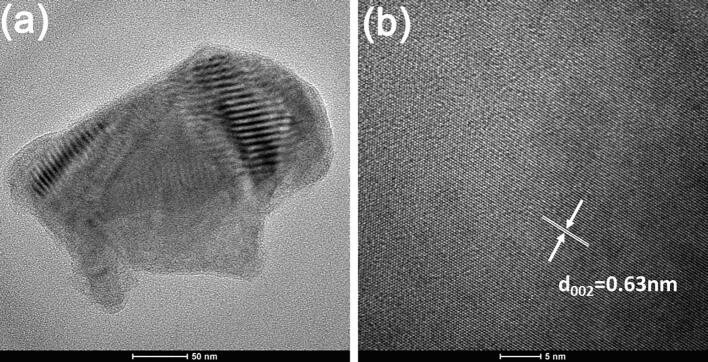
(a) TEM micrograph and (b) HRTEM image of an individual NbSe_2_ microplatet.

TEM micrograph and HRTEM image in Fig. 4 show that nanoplatets (Fig. 4a-b) and nanowhiskers are single crystal NbSe_2_ based on unit cell parameters in Fig. S2, which is also evident by Raman spectrum similar to in Fig. S1 and EDS in Fig. S3.

**Fig. 5:**
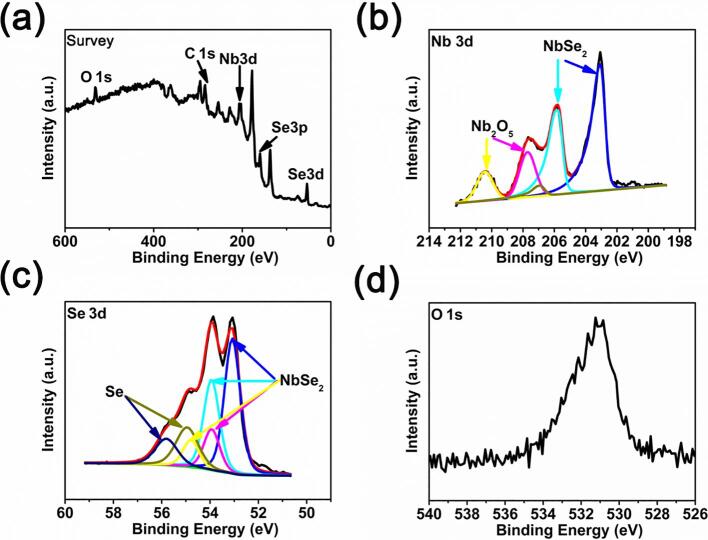
XPS spectra of (a) survey, (b) Nb3d, (c) Se3d, (d) O1s for ultrasonic-assisted exfoliated NbSe_2_ micro/nanoparticles.

XPS spectra in Fig. 5 show the presence of Nb_2_O_5_ and Se for ultrasonic-assisted exfoliated NbSe_2_ micro/nanoparticles. Nb_2_O_5_ and Se are the products of chemical reaction between NbSe_2_ micro/nanoparticles and ethanol. Nb_2_O_5_ can be removed by Ar^+^ ion sputtering for 60 s, see Fig. S4.

#### 3.2.2 With ageing

**Fig. 6:**
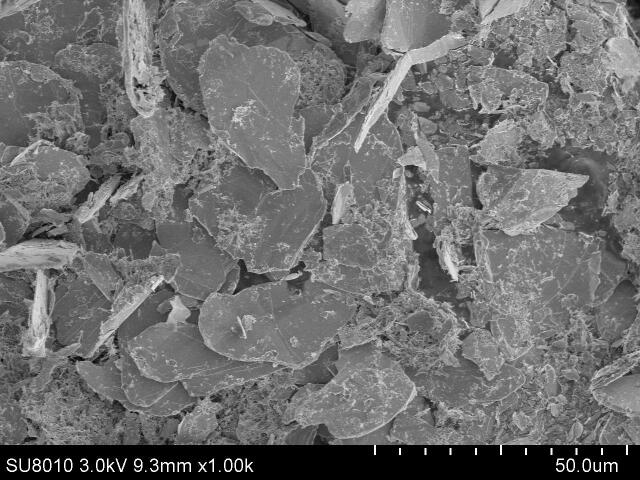
FESEM micrograph of ultrasonic-assisted exfoliated NbSe_2_ micro/nanoparticles after ageing for 15 days.

**Fig. 7:**
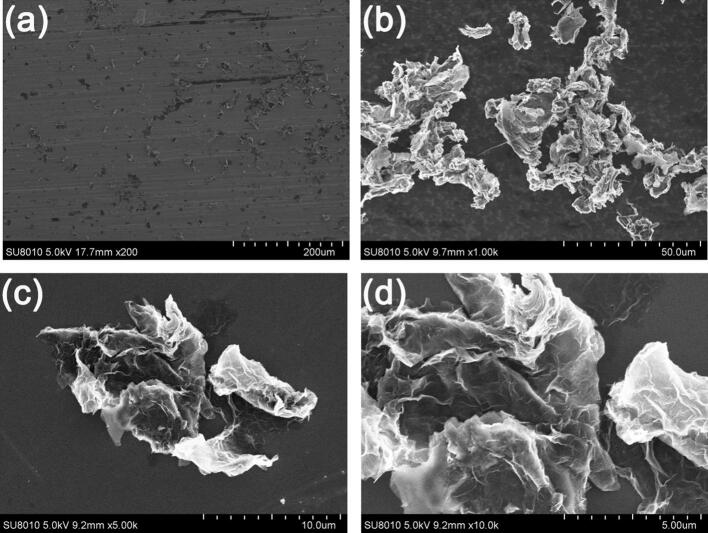
FESEM micrographs of (a) ultrasonic-assisted exfoliated NbSe_2_ micro/nanoparticles after ageing for 210 days and then deposited on a Cu disk by drop-casting, (b) floccules with a few nanowhisker and micro/nanoplatets, (c) agglomerated floccules, (d) corrugated floccules.

**Fig. 8:**
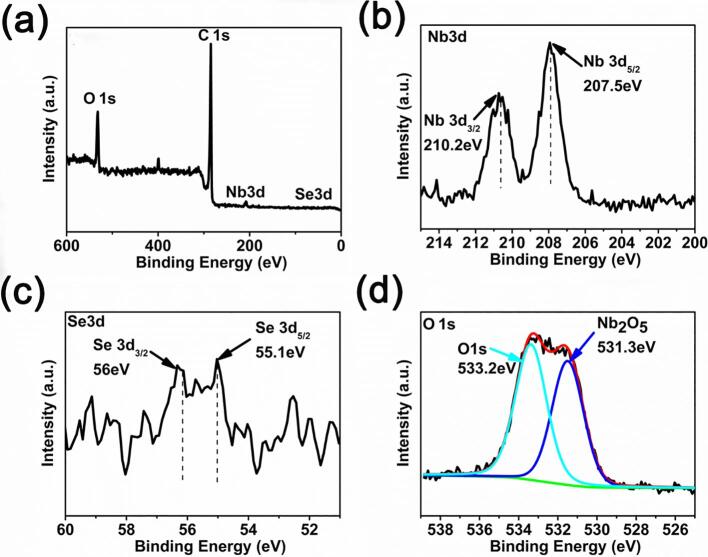
XPS spectra of (a) survey, (b) Nb3d, (c) Se3d, (d) O1s for ultrasonic-assisted exfoliated NbSe_2_ micro/nanoparticles after ageing for 210 days.

Short time ageing (e.g. 15 days) doesn’t obviously modify the morphology of NbSe_2_ micro/nanoparticles (Fig. 6) and XRD pattern (Fig. S2b) while prolonged ageing (e.g. 210 days) greatly modifies the morphology of NbSe_2_ micro/nanoparticles (Fig. 7) as well as the chemical composition by chemical interaction between NbSe_2_ micro/nanoparticles and ethanol (Fig. 8, Fig. S2c, Fig. S5). (0 0 2) plane of NbSe_2_ micro/nanoparticles after ageing for 210 days almost disappears in Fig. S2c. Elements C and O can also be found on NbSe_2_ micro/nanoparticles in Fig. S5. The drop-casting process enables a homogeneous distribution of micro/nanoparticles on the surface of Cu disk, see Fig. 7a. Most of the micro/nanoparticles are corrugated floccules (ca. 98%) with only a few nanowhisker (Fig. 7b) and a few micro/nanoplatets (Fig. 7b) are observed. Agglomeration of floccules can be seen in Fig. 7b and 7c. The morphology of corrugated floccules in Fig. 7d is similar to that of graphene nanoplatets.

**Fig. 9:**
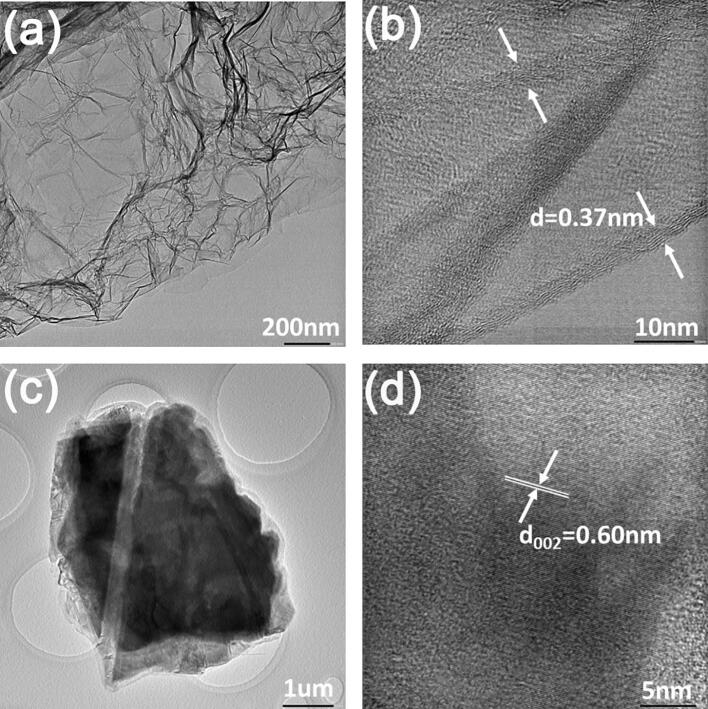
(a) TEM micrograph and (b) HRTEM image of floccules. (c) TEM micrograph and (d) HRTEM image of NbSe_2_ nanoplatets.

Chemical interaction between NbSe_2_ and ethanol is significant as evident by presence of Nb_2_O_5_, Se, and NbSe_2_ after ageing for 15 days. After ageing for 210 days, the peaks of Nb3d for NbSe_2_ vanishes and peaks of Nb3d for Nb_2_O_5_ dominates (Fig. 8a). This implies that NbSe_2_ will be totally consumed with prolonged time. HRTEM image suggests that corrugated floccules be composed of amorphous Nb_2_O_5_ and graphene (Fig. 9a-b) while NbSe_2_ micro/nanoplatets are composed of crystalline NbSe_2_ (Fig. 9c-d).

### 3.3 Exfoliation mode

As mentioned in section 2.1.2, the NbSe_2_ flake has ca. 2 × 2 × 0.5 mm in size, which means the flake is approximately composed of 60,000 to 80,000 layers. Mechanical exfoliation enables cleavage and delamination on 2H-NbSe_2_ flake and its fragments. NbSe_2_ microplatets in Fig. 1a has a thickness of ca. 2μ m (ca. 3000 layers) due to delamination along the basal plane and a plane size of 20 μm due to cleavage vertical to the basal plane (Fig. 1b, 3b).

The thickness of NbSe_2_ micro/nanoplatets is less than 1 μm, suggesting approximate 1500 layers. As a matter of fact, NbSe_2_ particles in ethanol suspension has a diversity of particle sizes, ranging from few-layer NbSe_2_ to micro/nanoplatets. Since the suspension is not centrifugated, the proportion of few-layer NbSe_2_ to micro/nanoplatets can not be determined.

Exfoliation mechanism under ball milling of NbSe_2_ powder suggests that nanosheet, nanobelt, nanorod, and nanoparticles by shear and plastic deformation be observed [23]. Cavitation-induced bubbles collapse and produce high temperature and high pressure which has impact on the delamination and cleavage of NbSe_2_ flake and its fragments. Fresh surface by delamination and cleavage are prone to chemical reaction with organic solvent. And this makes ageing a useful route to chemically modify the particles in the suspensions. Such a chemical modification may be accelerated by ageing at higher temperature and deserves further investigation.

### 3.4 Tribological property

**Fig. 10:**
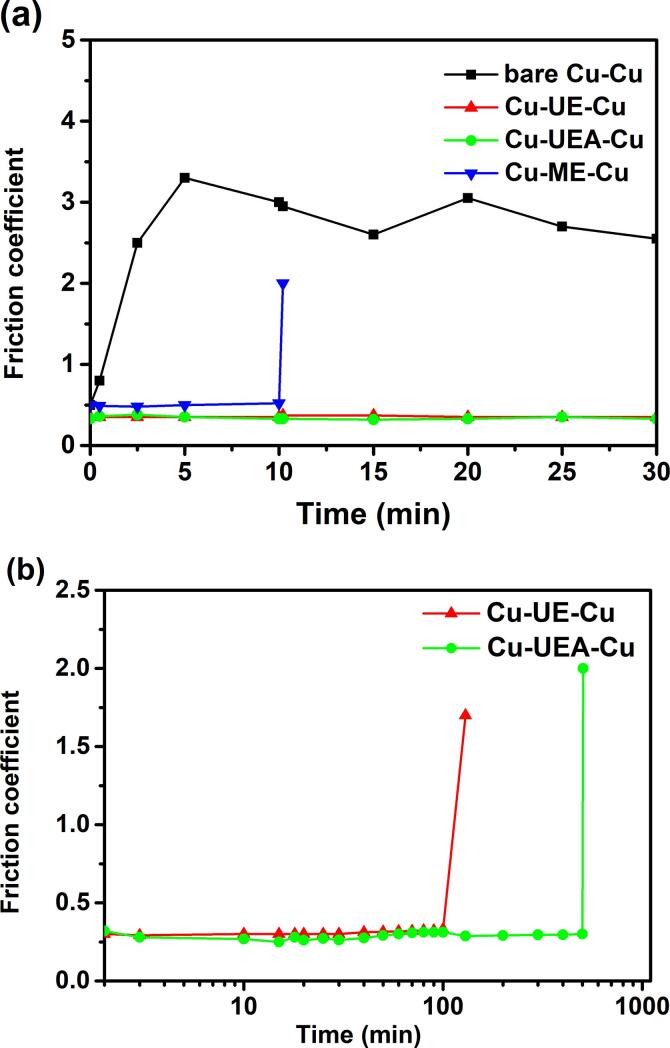
Frictional traces of (a) unlubricated Cu-on-Cu contact, mechanically exfoliated NbSe_2_ micro/particles. (b) NbSe_2_ micro/nanoparticles by ultrasonic-assisted exfoliation without and with ageing. ME: mechanically exfoliated; UE: ultrasonic-assisted exfoliation without ageing; UEA: ultrasonic-assisted exfoliation with ageing.

**Fig. 11:**
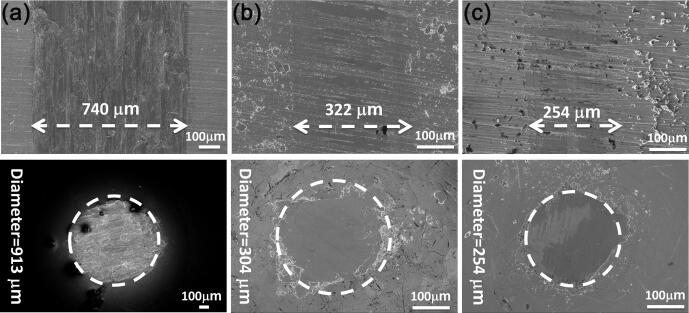
FESEM micrographs of worn surfaces of Cu disk (upper) and Cu pin (lower) in presence of NbSe_2_ microparticles by (a) mechanical exfoliation, (b) ultrasonic-assisted exfoliation without ageing, (c) by ultrasonic-assisted exfoliation with ageing.

Friction coefficient for unlubricated Cu-on-Cu contact is as high as 3.0 at steady stage (Fig. 10a), and severe adhesive wear and severe plastic flow on both Cu pin and Cu disk are observed (Fig. 11a). This is observed and explained by Antler [24]. In the presence of mechanically exfoliated NbSe_2_ micro/particles, friction coefficient is stable at ca. 0.5 for 10 min before tribological failure (a sudden increase in friction coefficient in Fig. 10a). NbSe_2_ micro/nanoparticles by ultrasonic-assisted exfoliation allow friction coefficient as low as ca. 0.3 and much longer wear lifetime, i.e. 120 min for one without ageing and 504 min for one with ageing, see Fig. 10b.

Typical characteristics of worn surface of “lubrication in good condition” are observed for NbSe_2_ micro/nanoparticles by ultrasonic-assisted exfoliation without ageing. These characteristics are: (1) lubricant film and original scratches on worn surface of Cu disk (upper micrograph in Fig. 11b) and (2) burnished surface on worn surface of Cu pin (lower micrograph in Fig. 11b). The same characteristics are also observed for NbSe_2_ micro/nanoparticles by ultrasonic-assisted exfoliation with ageing (Fig. 11c). The original parallel scratches are produced by abrading the Cu disk before drop-casting process. The preserved scratches indicates very mild wear on the Cu disk and the scratches act as the “reservoir” by trapping NbSe_2_ micro/nanoparticles (upper micrograph in Fig. 11b). It is very important because the amount of solid lubricant on the tribo-interface determines the wear lifetime of coatings of this kind. In other words, once NbSe_2_ on the worn surface is exhausted, tribological failure occurs.

The results of this work are compared with the previous work in Table 1. It should be pointed out that the friction coefficient reported in this study is the highest in Table 1. The friction coefficient of bare Cu-on-Cu contact is as high as 3, which is much higher than the reported values in references [1], [7], [8]. Friction coefficient of Cu-on-Cu contact in presence of NbSe_2_ micro/nanoparticles is merely one tenth of the friction coefficient of Cu-on-Cu contact in absence of NbSe_2_ micro/nanoparticles. In addition, NbSe_2_ micro/nanoparticles can also be used as a solid lubricant for running-in procedure of a brush/slip assembly.

**Table 1:** Comparison on tribological property of NbSe_2_ as solid lubricant with previous literature reports.

Material and process	Friction coefficient	Wear rate or wear regime	Refs.
NbSe_2_ coating (1.4 and 1.6 μm in thickness) with a Ti intermediate layer by radio frequency magnetron sputtering	<0.1	10^−15^ m^3^N^−1^m^−1^	[8]
Ag-NbSe_2_ composite by powder metallurgy	0.2	10^−14^ m^3^N^−1^m^−1^	[1]
Cu-NbSe_2_ composite by powder metallurgy	0.15–0.20	N/A	[7]
NbSe_2_ micro/nanoplatets (8% coverage on the worn surface) by drop-casting	0.3	Mild wear, 10^−15^ m^3^N^−1^m^−1^ before worn out	This study

## 4 Conclusions

A top-down preparation of NbSe_2_ micro/nanoparticles by mechanical exfoliation and ultrasonic-assisted exfoliation in ethanol without and with ageing. Ultrasonic-assisted exfoliation produces NbSe_2_ micro/nanoplatets and nano-whiskers, as well as Nb_2_O_5_ and Se. Prolonged ageing of the suspensions modifies the morphology by converting platets and whiskers into corrugated floccules, which are composed of Nb_2_O_5_, Se, and graphene. NbSe_2_ micro/nanoparticles by ultrasonic-assisted exfoliation allow sliding with low friction coefficient (0.30), mild wear (wear too low to measure). The wear lifetimes in presence of NbSe_2_ micro/nanoparticles (120 min for the in-aged one and 504 min for the aged one) are much longer that of mechanical exfoliation (10 min). The profound advantages in easy deposition on Cu substrate, good tribological property promise NbSe_2_ micro/nanoparticles to be good solid lubricant for sliding electrical contact. NbSe_2_ micro/nanoparticles can also be used as solid lubricant for running-in procedure of a brush/slip assembly.

## CRediT authorship contribution statement

**Rong Qu:** Methodology, Investigation, Data curation. **Xiaoqin Wen:** Investigation, Data curation, Writing - original draft. **Yamei Zhao:** Formal analysis, Investigation. **Tingmei Wang:** Methodology, Validation. **Ruiqing Yao:** Writing - review & editing. **Jinjun Lu:** Supervision.

## Declaration of Competing Interest

The authors declare that they have no known competing financial interests or personal relationships that could have appeared to influence the work reported in this paper.
