# Supplementary material for: Ultrasonic-assisted top-down preparation of NbSe2 micro/nanoparticles and hybrid material as solid lubricant for sliding electrical contact
Source: Ultrason Sonochem. 2021 Feb 10;73:105491. doi: 10.1016/j.ultsonch.2021.105491 (PMC7902518; doi:10.1016/j.ultsonch.2021.105491)
Supplement: Supplementary data 1 [file mmc1.doc]

**Supporting information**


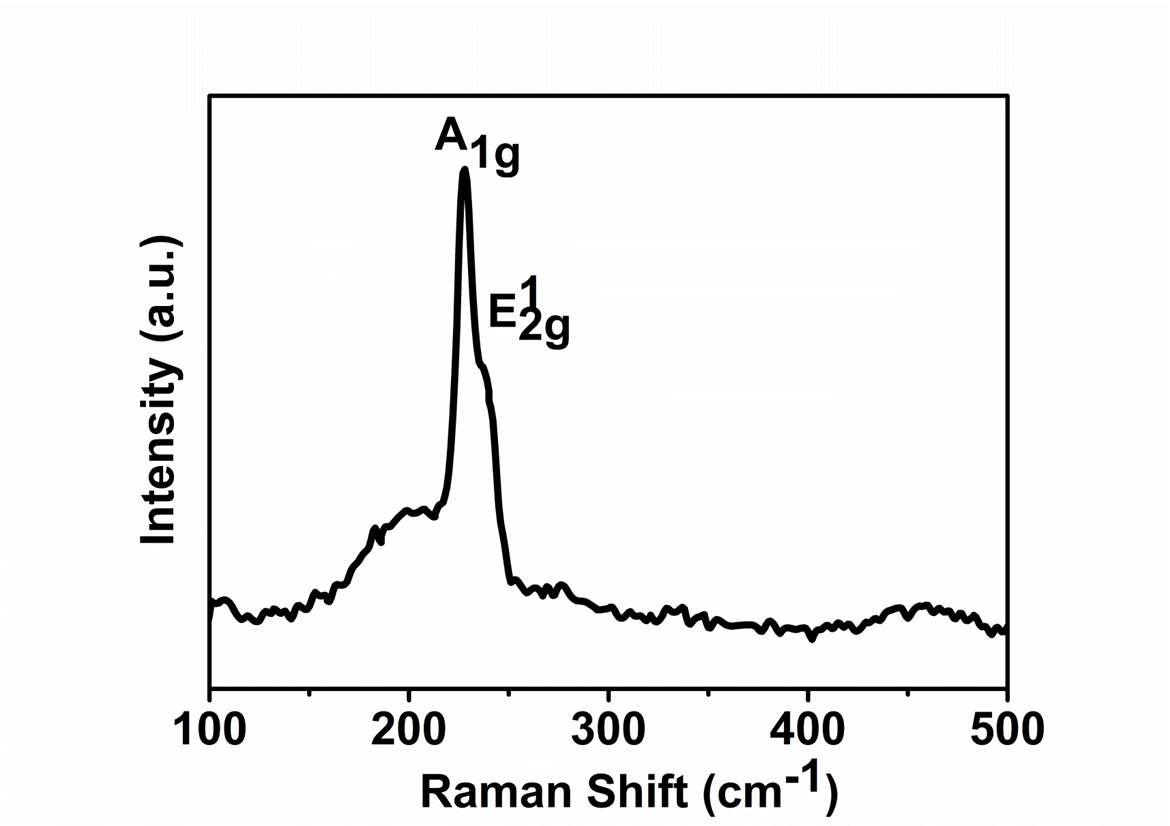


Fig. S1 Raman spectrum of NbSe_2_ microplatets deposited on a T2 Cu disk.


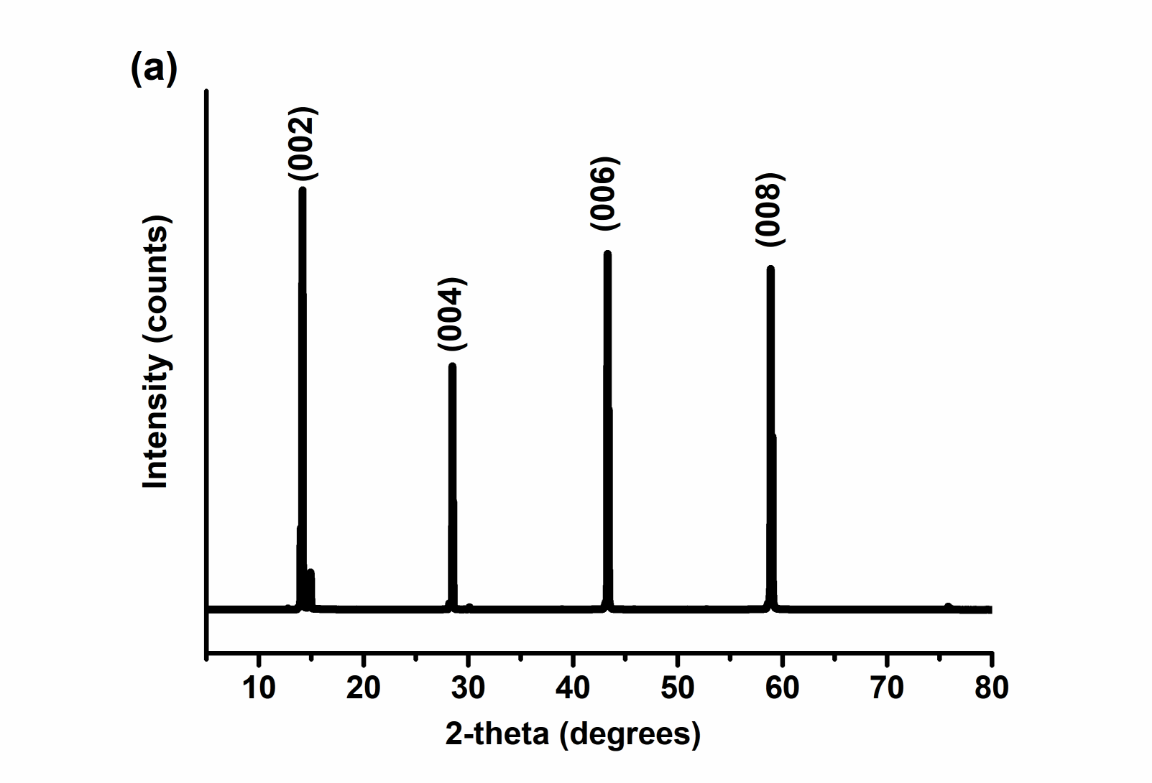

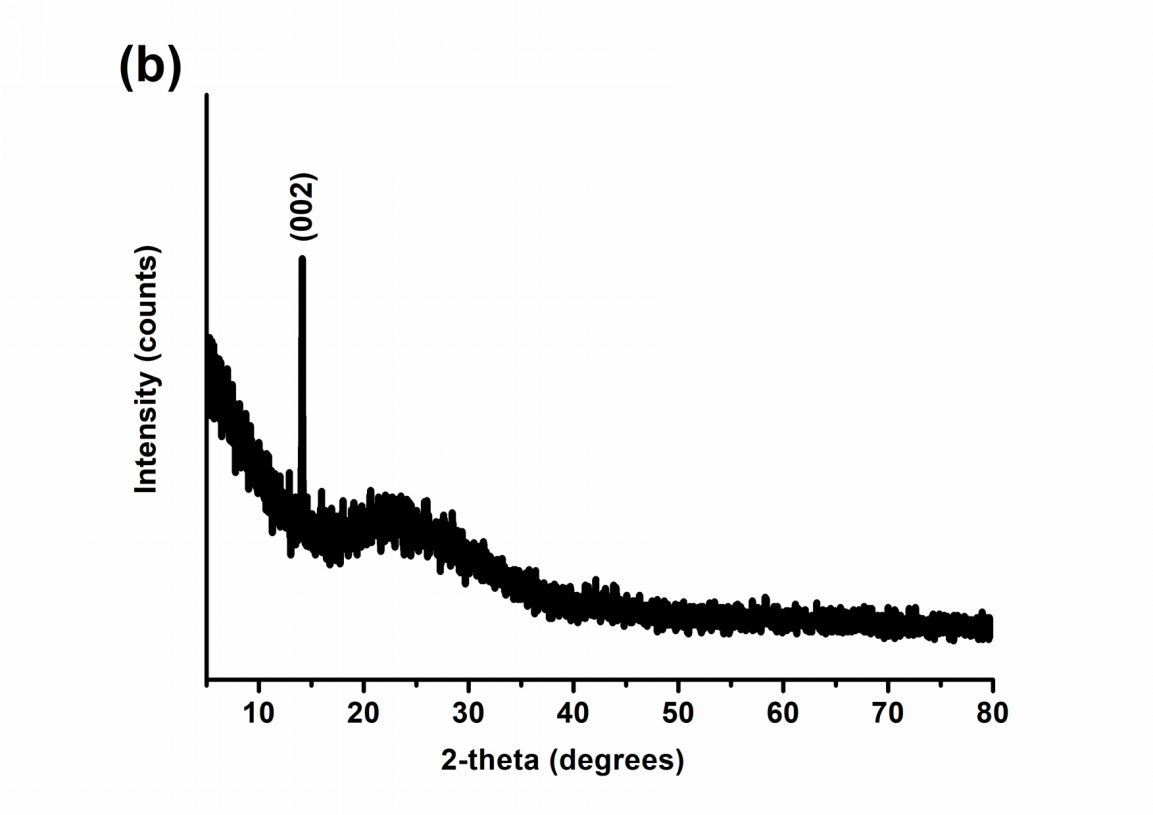


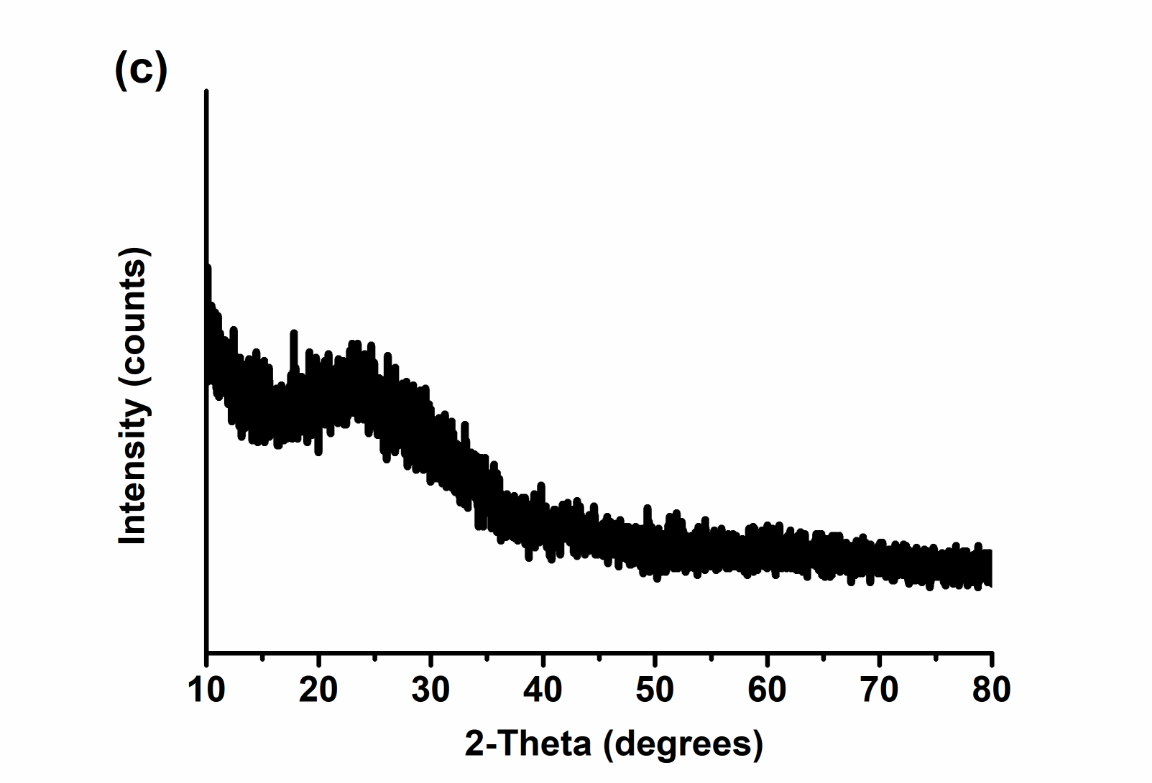


Fig. S2 XRD patterns of (a) 2H-NbSe_2_ flake, (b) NbSe_2_ micro/nanoparticles by ultrasonic-assisted exfoliation after ageing for 15 days, (c) NbSe_2_ micro/nanoparticles by ultrasonic-assisted exfoliation after ageing for 210 days. According to PDF#65-7289, NbSe_2_ has a crystal structure as hexagonal and a space group of P63/mmc(194). Unit cell parameters, a = b = 0.344 nm, c = 1.255 nm, α = β = 90°, γ = 120° can also be obtained from pdf document.


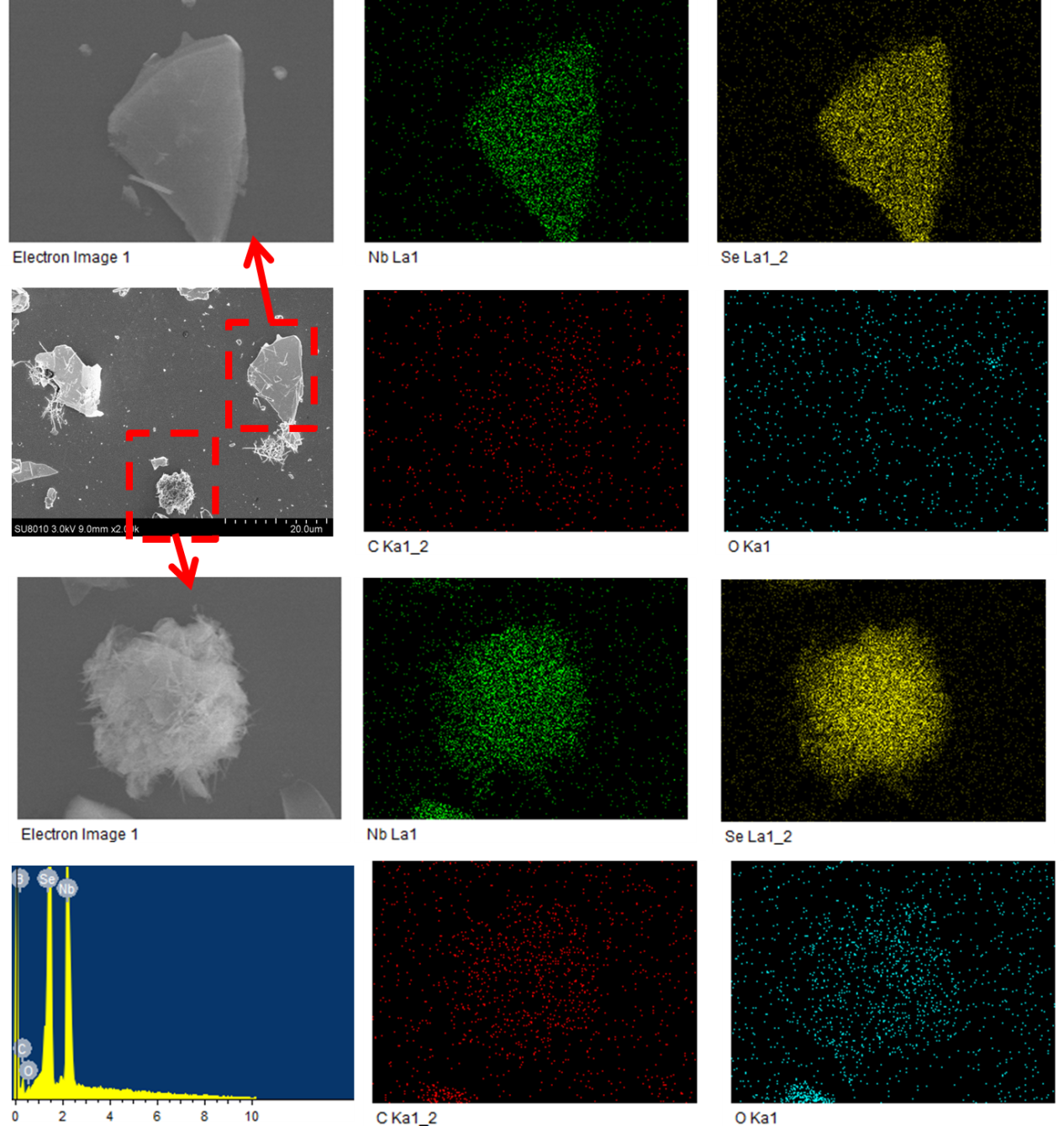


Fig. S3 FESEM micrograph, elemental mapping and EDS spectrum of ultrasonic-assisted exfoliated NbSe_2_ micro/nanoparticles without ageing.





Fig. S4 XPS spectra of Nb3d for ultrasonic-assisted exfoliated NbSe_2_ micro/nanoparticles before and after sputtering.


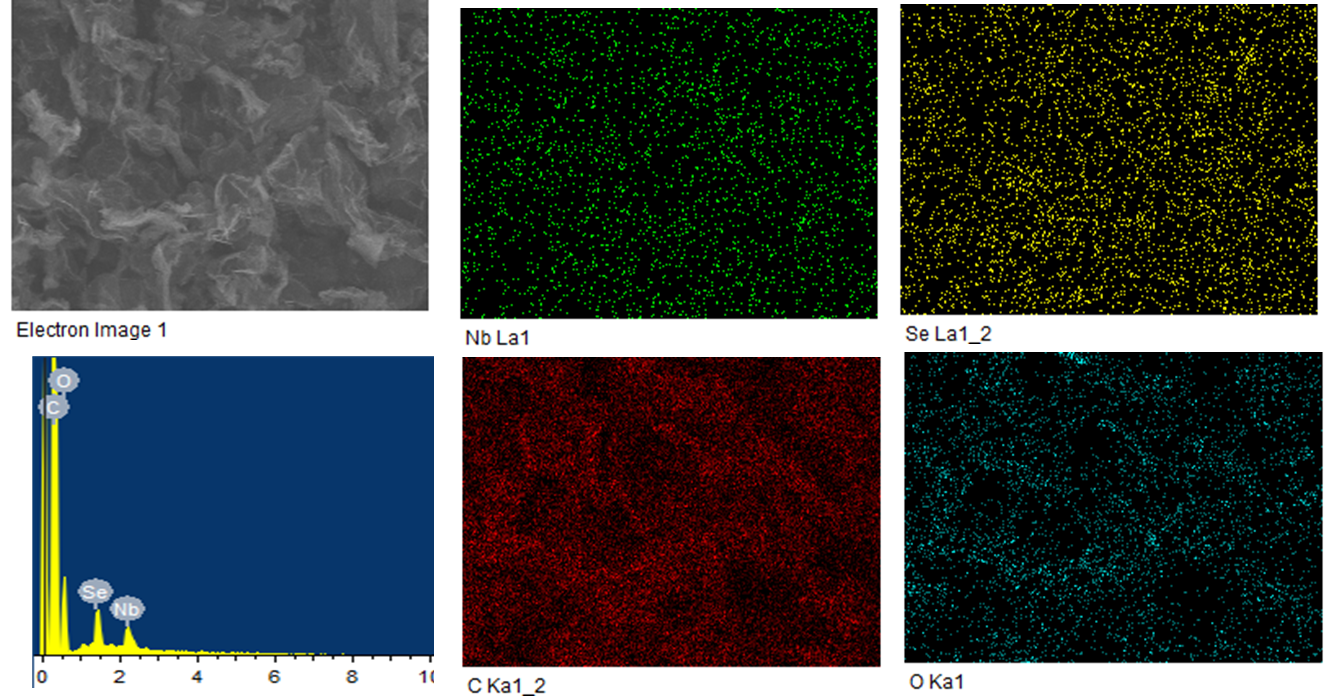


Fig. S5 FESEM micrograph, elemental mapping and EDS spectrum of ultrasonic-assisted exfoliated NbSe_2_ micro/nanoparticles after ageing for 210 days.
